# Construction of Biomimetic Film Based on the Surface Structure of Orange Peel and Its Blueberry Preservation Performance

**DOI:** 10.3390/gels12070573

**Published:** 2026-06-29

**Authors:** Xiuqi Liu, Xingyu Chen, Feiyao Wang, Yixuan Zhang, Mingxing Li, Daoyin Zhang, Jing Qiao, Liyan Wang, Lili Ren

**Affiliations:** 1 College of Food Science and Engineering, Jilin Agricultural University, Changchun 130118, China; xiuqil@jlau.edu.cn (X.L.); c1565472774@outlook.com (X.C.); wangfeiyao1215@163.com (F.W.); zyx_jlau@163.com (Y.Z.); limingxing@jlau.edu.cn (M.L.); 20221087@mails.jlau.edu.cn (D.Z.); jlau010406@163.com (J.Q.); 2 Key Laboratory of Bionic Engineering, Ministry of Education, Changchun 130022, China

**Keywords:** orange peel, bionic, bioactive gel film, surface structure, blueberry, preservation

## Abstract

To develop eco-friendly and highly efficient fruit and vegetable preservation materials, this study uses the multi-gradient micro–nano roughness structure and bioactive properties of orange peel as a biomimetic model, aiming to construct a functional film with a unique dual mechanism of physical barrier protection and active preservation. Using soft etching and secondary transfer methods, with polydimethylsiloxane as an intermediate template, and through a repeated freeze–thaw cross-linking process, a polyvinyl alcohol system containing orange peel essential oil was cast to successfully prepare a biomimetic film featuring the micro–nano hierarchical structures found on the surface of orange peel. The study indicates that the biomimetic film accurately replicates the cross-scale hierarchical structures of the natural orange peel surface. Structure–property relationship analysis revealed that the biomimetic film containing 15% orange peel essential oil exhibited the optimal comprehensive performance, characterized by significantly enhanced tensile strength and improved water vapor barrier properties, while demonstrating effective antioxidant and regulated antibacterial activities. Crucially, compared to conventional flat active films, the replicated multi-scale surface roughness provides clear functional advantages by physically optimizing interface properties and cooperating synergistically with the chemical vapor release of the essential oil. Blueberry preservation experiments confirmed that the biomimetic film successfully maintains fruit firmness, vitamin C, and anthocyanin content, while suppressing weight loss and decay rates. This study simulates the microenvironmental control mechanisms of orange peel, highlighting the scientific novelty of structural–chemical synergistic design for advanced functional packaging.

## 1. Introduction

As the primary natural barrier protecting orange fruits from adverse environmental conditions and pathogenic infections, the complex biological structure of orange peel provides an excellent protective mechanism. Studies indicate that the orange peel surface is covered by a unique topography formed by the interplay between epicuticular wax and micro-scale hierarchical structures, resulting in a dense, irregular micro-porous network [1]. This multi-scale rough structure provides physical cushioning and mechanical support for the internal flesh [2,3]. However, during orange consumption and industrial processing, this tissue is predominantly discarded as a byproduct. Due to high moisture and sugar concentrations, traditional landfill disposal easily triggers decay, causing severe environmental pollution and resource waste [4].

Blueberries are rich in antioxidant compounds like vitamin C and possess a wide range of medicinal benefits [5,6], often hailed as the “king of berries” [7]. However, due to their thin skin, soft texture, and high water content, they are highly susceptible to spoilage [8,9]. Therefore, extending their postharvest storage life is imperative to minimize nutritional losses and support the blueberry industry [10]. Recently, biological preservation technologies have gained widespread favor due to their eco-friendly, efficient, and safe characteristics, becoming a major research hotspot [11]. Previous studies developed various safe bio-preservation films, such as a chitosan/enoki mushroom root polysaccharide composite film that slows blueberry spoilage [12] and a chitosan/whey protein isolate composite film that extends strawberry shelf life by 5 to 8 days [13]. Nevertheless, critical limitations persist in these traditional active packaging platforms. Conventional composite films based on biopolymer blends inherently lack a physical anti-adhesion barrier due to their flat, featureless macro-surfaces, making them highly susceptible to microbial boundary colonization under high-humidity conditions. To address these bottlenecks, biomimetic material science has injected fresh vitality into this domain by emulating the sophisticated structures of flora and fauna. For instance, researchers have replicated the porous “gas switches” of plant leaves using shellac and chitosan, or engineered micro-patterned surfaces mimicking insect wings to induce physical damage to microbial cell membranes [14]. However, most current biomimetic patterns rely on high-cost lithography, complex physical/chemical etching, or toxic modification reagents, hindering their scalability. Furthermore, previous studies on microstructured films are largely descriptive regarding topographical replication, frequently overlooking the critical interfacial interplay between surface physical roughness and internal active preservation agents. While dual-functional films have been developed by mechanically incorporating synthetic silver nanoparticles or external waxy layers, a strategy fully exploiting a single natural waste-derived biomass as a homologous template remains unexploited [15]. Therefore, developing a cost-effective, eco-friendly biomimetic film that concurrently dictates both the physical barrier and the active chemical microenvironment through a single biomass framework presents a major scientific challenge.

This study used orange peel as a biomimetic model and polydimethylsiloxane (PDMS) as a substrate to obtain a PDMS anti-structure negative film; using polyvinyl alcohol (PVA) as the substrate and orange peel essential oil (OPEO) as the active ingredient, a PVA pro-structure positive film (OPEO/PVA biomimetic film) with the micro- and nanostructures of orange peel was prepared. The study investigated the preparation methods and characterization of polymer biomimetic films with cross-scale hierarchical gradient structures. By examining the structural and physicochemical properties of the biomimetic films, and evaluating the preservation capacity of the OPEO/PVA biomimetic film for blueberries stored at 4 °C for 20 days, the research aims to develop biomimetic films that are both environmentally friendly and safe, while possessing antibacterial and preservative functions. Furthermore, it seeks to provide new insights for the development of novel biocompatible biomimetic materials.

## 2. Results and Discussion

### 2.1. Microstructures of Anti-Structural Films and Biomimetic Films

**Figure 1:**
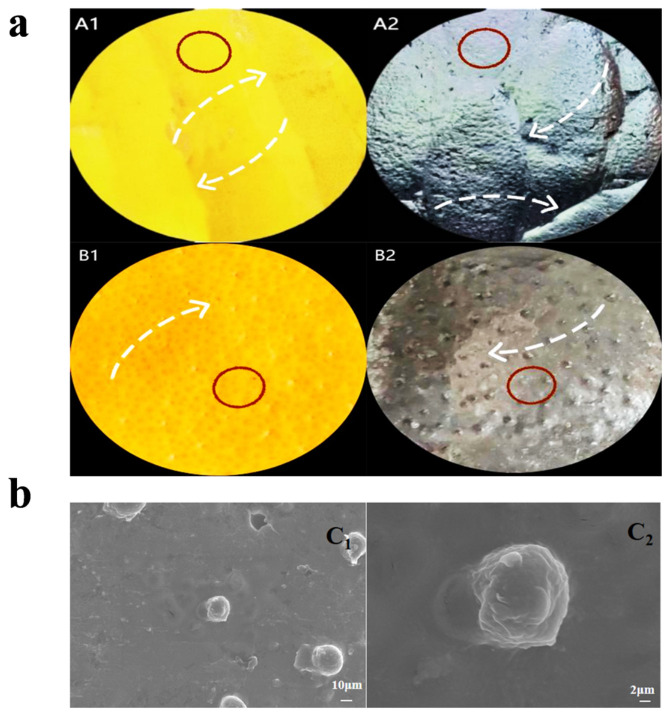
(**a**) Orange-peel-like surface structure (microscope images A1, B1) and SP-PDMS film surface structure (microscope images A2, B2). (**b**) Scanning electron microscope images of the biomimetic film (BPO-15 group). In figure (**a**), the white dashed arrows denote the macro-texture orientation or replication directions, and the red solid circles highlight the replicated microstructures (e.g., oil glands or stomata-like pores). In figure (**b**), C1 and C2 represent the SEM surface views at different magnifications.

Both the orange peel surface and the anti-structured polydimethylsiloxane (SP-PDMS) film consist of alternating rows of protrusions and grooves, as shown in Figure 1a. The grooved structure on the orange peel surface constitutes its primary structure (see arrows in Figure 1a(A1,A2)); the closely packed, smooth surface of the orange peel represents the secondary structure, which exhibits a regular, extended distribution (see circled area in Figure 1), terminating in submicron-scale tertiary-level protrusions (see arrows in Figure 1a(B1,B2)). The grooves, smooth surfaces, and protrusions overlap to form the unique micro–nano structure of the orange peel surface. The SP-PDMS film successfully replicates the primary, secondary, and tertiary structures of the orange peel model. As shown in the SEM images (Figure 1b), the OPEO-loaded biomimetic films preserved the original hierarchical micro–nano features of orange peel, demonstrating that OPEO loading had no adverse effect on structural integrity. In particular, the BPO-15 film exhibited a smooth and compact surface without observable phase separation or oil droplet aggregation. The highly matched morphological features among natural orange peel, PDMS negative mold, and BPO films in Figure 1 demonstrated excellent surface fidelity and replication efficiency. This intact structure transfer was inherited from the nanometer-scale replication precision naturally provided by the PDMS soft lithography process. Although the qualitative SEM and optical microscopy images successfully confirm the intact replication of the cross-scale hierarchical bionic structures, a precise 3D numerical mapping of surface roughness parameters (such as Ra, Rq, or Sa) was not conducted at this stage. These quantitative topographical metrics are critical for deeply understanding the micro-scale interactions at the film interface. While the current visual evidence firmly supports the presence of multi-scale gradient roughness, establishing exact mathematical correlations via AFM or profiling techniques remains a limitation of this study, which will be prioritized in our future work.

### 2.2. Analysis of the Physical Properties of Biomimetic Films

**Figure 2:**
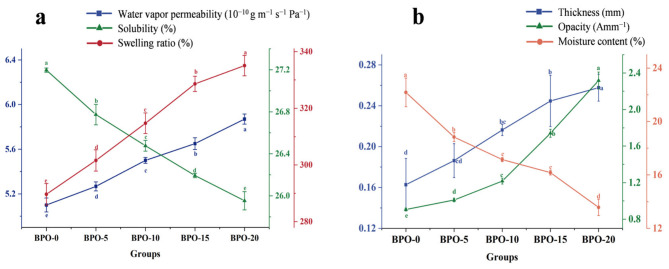
Physical properties of biomimetic films from different orange peel essential oil groups. (**a**) Water vapor permeability, swelling ratio, and solubility of biomimetic films containing different concentrations of orange peel essential oil. (**b**) Thickness, opacity, and water content of biomimetic films. Each value represents the mean ± standard deviation (*n* = 3). Different lowercase letters (ae) above the bars indicate significant differences (*p* < 0.05) among groups, determined by one-way ANOVA followed by Duncan’s multiple range test.

As illustrated in Figure 2a, the water vapor permeability (WVP) of the biomimetic films exhibited a progressive upward trend with increasing orange peel essential oil (OPEO) concentration. The incorporation of OPEO likely disrupted the original intermolecular hydrogen bonding within the PVA matrix, leading to a looser network structure that facilitated water vapor diffusion (*p* < 0.05), consistent with the findings of Wai et al. [16]. This hypothesis of network loosening is directly and robustly supported by the FTIR and XRD structural data in Section 2.3. The shifting of O-H bands combined with the collapse of crystallinity from 44.26% to below 3.0% firmly validates that OPEO molecules successfully disrupted the dense crystalline packing of PVA, creating larger free volume for water vapor migration.

Similarly, the swelling degree of the biomimetic films increased progressively with higher OPEO concentrations. Although the hydrophilic nature of the hydroxyl groups in PVA remains the dominant factor governing water affinity, the OPEO-induced reduction in cross-linking density facilitated water penetration into the loosening polymer network, thereby promoting structural expansion [17,18]. In contrast, both the water solubility and moisture content (MC) exhibited a negative correlation with OPEO content. Specifically, the significant decrease in solubility and the reduction in MC (from 22.18% to 13.57%, *p* < 0.05) can be attributed to the inherent hydrophobicity of OPEO, which enhances the film’s resistance to water dissolution while simultaneously diminishing its overall water-retaining capacity [19].

When utilized as a food wrap, the transparency of the film allows users to directly assess the freshness of the wrapped items, thereby facilitating the convenient monitoring of food conditions [20]. As shown in Table 1 and Figure 2b, increasing the orange peel essential oil (OPEO) content significantly altered the optical properties of the biomimetic films. The total color difference (E*) increased significantly (*p* < 0.05), while the L* values and transparency decreased. This trend aligns with the significant rise in opacity, which escalated from 0.91 Amm^−1^ (BPO-0) to 2.32 Amm^−1^ (BPO-20). Interestingly, the stable and well-controlled E* values observed in the BPO-20 group indicate enhanced color uniformity at higher OPEO concentrations. These optical modifications are primarily driven by D-limonene, the major component of OPEO; its pale yellow hue and potential conjugated double-bond systems dominate the yellow chromaticity of the film (reflected in the a* and b* values) while simultaneously obstructing light passage [20,21].

In contrast, the thickness of the biomimetic films showed a significant upward trend (*p* < 0.05). Compared to BPO-0 (0.16 mm), the thickness of BPO-15 and BPO-20 increased to 0.24 mm and 0.26 mm, respectively—representing increases of 50.0% and 62.5%. Quantitatively, the 50.0% and 62.5% increase in thickness for BPO-15 and BPO-20 precisely matches the upward trend of the swelling degree (Figure 2a). This indicates that the volume expansion is co-driven by the plasticized expansion of the amorphous PVA matrix and the structural occupation of embedded OPEO droplets within the hydrogel voids, which mechanically forces the network to swell outward [22].

### 2.3. Structural Characterization

**Figure 3:**
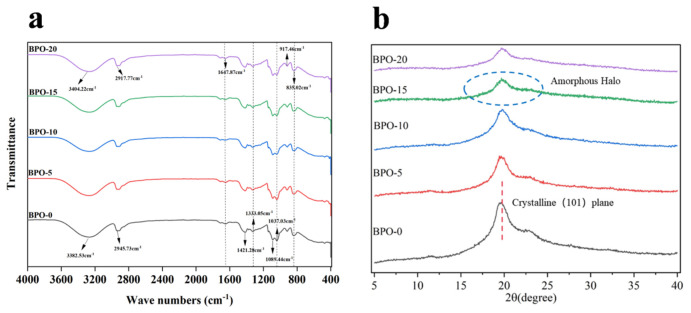
Analysis of the microstructure of biomimetic film. (**a**) Fourier infrared spectra. (**b**) XRD of the bionic films (Red dashed line represents the crystalline (101) plane peak; blue dashed circle highlights the amorphous halo).

As shown in Figure 3a, the BPO-0 group exhibits peaks at 3382.55 cm^−1^ (O-H stretching), 2945.73 cm^−1^ (C-H stretching), 1421.28 cm^−1^ (C-H bending), 1333.05 cm^−1^ (O-H in-plane bending), and 1089.44/1037.03 cm^−1^ (C-O stretching), which is consistent with the structure of PVA [23]. With the addition of orange peel essential oil, the O-H stretching peak of PVA shifted to a higher wavenumber (3404.22 cm^−1^ for the BPO-20 group), indicating the alteration of the hydrogen-bonding network between PVA and the orange peel essential oil. This chemical shift can be fundamentally attributed to the presence of D-limonene, the dominant hydrophobic monoterpene in OPEO. Although D-limonene lacks hydroxyl groups, its bulky hydrocarbon structure inserts into the PVA matrix, generating intensive local hydrophobic interactions and steric hindrance. This spatial incorporation interrupts the original self-associated intermolecular hydrogen bonds of pure PVA, leading to a reorganized network and the subsequent migration of O-H bands. Concurrently, new peaks appeared in the biomimetic film at 1647.87 cm^−1^ (C=C stretching vibration of terpenes), 917.46 cm^−1^ and 835.02 cm^−1^ (out-of-plane bending vibrations of the C-H bond in terpenes), and their intensities increased with the increase in orange peel essential oil content. The surface structure of the orange peel had no effect on the blending of polyvinyl alcohol and orange peel essential oil; the two exhibited good compatibility.

Figure 3b shows the X-ray diffraction patterns of the various groups of biomimetic films. The absorption peak at 2θ = 19.8° in the BPO-0 group, which did not contain sweet orange essential oil, was sharp and high, indicating a high degree of crystallinity. With the addition of sweet orange essential oil, the intensity of the peak at 2θ = 19.8° decreased. For the BPO-0 group (without orange peel essential oil), a sharp and intense crystalline diffraction peak appeared at 2θ = 19.8°, characteristic of the (101) plane of PVA crystals. With increasing essential oil content, this sharp peak gradually weakened. As shown in Table 2, in the BPO-15 group, the crystalline peak completely vanished and was replaced by a broad, low-intensity amorphous halo centered at approximately 19.8° (full width at half maximum > 5°), indicating a predominantly amorphous structure. This transition from a sharp crystalline peak to a broad amorphous halo at a similar 2θ position occurs because the average intermolecular distance in amorphous PVA is close to the (101) d-spacing of its crystalline phase [24]. At the same time, the absorption peak at 2θ = 19.8° for the BPO-20 group is similar to that of the BPO-15 group. This may be because the excess sweet orange essential oil could not bind to the PVA, resulting in the absorption peaks of the two groups being similar [25]. As clearly quantified in Table 2, the crystallinity index (Xc) of the films exhibited a drastic, stepwise reduction upon OPEO incorporation, collapsing from 44.26 ± 0.65% (BPO-0) to merely 2.31% (BPO-15). This sharp amorphization trend captured in Table 2 serves as the structural foundation for explaining the mechanical performance reported in Table 3. The nearly complete elimination of the dense crystalline domains significantly liberated the constraints on the PVA backbone segments. This microstructural transition directly accounts for the matrix relaxation, which perfectly justifies the continuous drop in tensile strength and the remarkable enhancement in elongation at break (EAB).

### 2.4. Mechanical Properties of Biomimetic Films

Mechanical properties are crucial for evaluating the practical applicability of packaging films. Specifically, tensile strength (TS) and elongation at break (EAB) represent a film’s resistance to tearing and its ductility, respectively. Superior mechanical properties are essential for maintaining film integrity against external forces, thereby protecting packaged food from external contamination and ensuring food safety [26]. To systemically evaluate the influence of varying OPEO concentrations on these characteristics, the TS and EAB of the biomimetic films were comprehensively quantified.

Table 3 shows that adding OPEO caused the tensile strength of the biomimetic films to first increase and then decrease. The BPO-15 group reached the maximum value (7.65 ± 0.22 MPa), approximately 43.3% higher than the BPO-0 group. This enhancement is due to hydrogen bonding between PVA hydroxyl groups and OPEO functional groups, which promotes a tighter, more ordered molecular arrangement and strengthens matrix cohesive forces [27]. However, at 20% OPEO, excess oil cannot fully integrate into the polymer matrix, leading to reduced load-bearing capacity and lower tensile strength.

The elongation at break of the biomimetic films first increased and then decreased with higher orange peel essential oil (OPEO) content. The BPO-5 group exhibited the maximum elongation at break (174.32 ± 7.48%), a 13.99% increase over BPO-0, indicating peak ductility. This initial improvement suggests that low OPEO doses exert a plasticizing effect on PVA segments, enhancing chain flexibility and ductility. Critically, as the OPEO concentration further elevated to 20% (BPO-20), a distinct reduction in EAB was observed. This inversion can be fundamentally explained by the micro-phase separation threshold of the composite matrix. Although essential oils generally serve as plasticizers at lower loadings, an overdose of hydrophobic D-limonene exceeds the compatibility limit of the hydrophilic PVA networks. During the freeze–thaw crystallization process, the unbonded oil domains tend to undergo localized aggregation, forming microstructural oil pockets. These heterogeneous domains act as severe stress concentration points within the cross-linked network. Under external tensile stress, micro-cracks propagate prematurely from these weak interfacial boundaries, inducing catastrophic failure prior to full chain extension, which macroscopically manifests as a compromised elongation capability [28]. Importantly, the overall tensile strength (ranging from 4.79 to 7.65 MPa) and outstanding elongation at break (up to 174.32%) of the developed BPO biomimetic films are highly competitive with recently reported active packaging films containing essential oils or plant extracts [29].

### 2.5. Bioactive Properties of Biomimetic Films

As summarized in Table 4, the incorporation of OPEO significantly enhanced the DPPH and ABTS radical scavenging capacities of the biomimetic films (*p* < 0.05). The antioxidant activity exhibited a concentration-dependent increase, though the rate of enhancement slowed in the BPO-20 group, identifying BPO-15 as the optimal formulation for balanced performance. This high activity is attributed to the synergistic effect between the OPEO and the bionic surface topography. Specifically, the coarse-graded biomimetic structure inherited from the orange peel enhances oxygen barrier properties, while the active polyphenols and flavonoids within the OPEO effectively neutralize oxidative species [30].

The antibacterial efficacy of the biomimetic films against *Escherichia coli* and *Staphylococcus aureus* is presented in Table 4. While the BPO-0 group showed no inhibitory effect, the OPEO-loaded groups exhibited a moderate but statistically significant increase in their inhibition zones (*p* < 0.05). The BPO-20 group demonstrated the largest inhibition diameters of 8.40 mm for E. coli and 7.30 mm for S. aureus, indicating that the developed biomimetic films exert a regulated yet effective growth inhibition against both Gram-negative and Gram-positive strains. To better understand this performance, the mass transfer of the active volatiles from the matrix to the surrounding medium should be evaluated. Mechanistically, the migration of hydrophobic OPEO components (predominantly D-limonene) out of the polymeric structure is governed by a moisture-induced swelling-controlled diffusion mechanism. Upon contact with the humid interface of the agar medium, water molecules rapidly penetrate and plasticize the hydrophilic PVA matrix, provoking network expansion and enlarging the fractional free volume. This structural relaxation unlocks the embedded oil pathways, enabling a steady, sustained Fickian diffusion rather than an instantaneous burst release [31,32]. Consequently, although the resulting zone of inhibition under immediate in vitro observation remains moderate due to this controlled release, this continuous, steady-state diffusion profile is highly advantageous for active packaging as it ensures a prolonged preservation headspace. Furthermore, the multiscale hierarchical gradient structure of the bionic surface increases surface roughness, which physically interferes with bacterial adhesion and boundary colonization [33], working synergistically with the chemical toxicity of OPEO. While direct MIC/MBC values and in vitro assessments against specific blueberry spoilage fungi (such as Botrytis cinerea) were limited at this current stage, the practical efficacy of this dual chemical–physical antimicrobic barrier is fully substantiated by the highly suppressed mold decay during the 20-day real fruit preservation trials discussed in Section 2.6.

In summary, BPO-15 (15% OPEO) was identified as the optimal formulation due to its excellent compatibility and its ability to closely mimic natural orange peel. Based on these comprehensive structure–activity relationships, the entire series of biomimetic films (BPO-0 to BPO-20) was subsequently tested in blueberry preservation trials to evaluate their effectiveness in maintaining fruit quality and nutritional value compared to an unpackaged control group.

### 2.6. Application of Bio-Inspired Film in Blueberry Preservation

**Figure 4:**
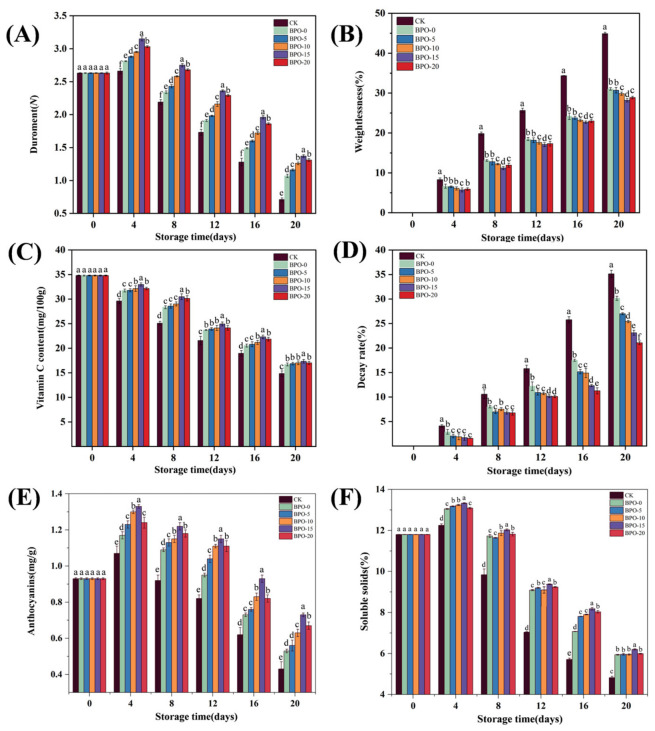
Effects of different biomimetic films on the physicochemical and nutritional properties of blueberries during storage. (**A**) Firmness (Durometer); (**B**) weight loss rate; (**C**) Vitamin C contents; (**D**) decay rate; (**E**) anthocyanin contents; (**F**) soluble solids. Note: Data are presented as mean ± standard deviation (*n* = 3). Different lowercase letters (a–f) above the bars within the same storage day indicate significant differences between groups (*p* < 0.05) according to Duncan’s multiple range test.

Firmness, as quantified by a durometer, serves as a pivotal indicator of blueberry post-harvest quality [34]. As shown in Figure 4A, all groups exhibited an initial increase followed by a decline; however, the BPO-15 group maintained the highest structural retention (1.37 N) by day 20, significantly outperforming the CK group (*p* < 0.05). This preservation is primarily attributed to the biomimetic films’ efficacy in restricting oxygen permeation, which suppresses respiratory intensity and delays the enzymatic degradation of cell wall pectin [35]. Conversely, the unpackaged CK group underwent rapid softening due to uncontrolled metabolic activity.

Post-harvest transpiration and respiration are the primary drivers of moisture loss and nutrient depletion in blueberries [36]. As shown in Figure 4B, the weight loss rate increased in all groups, with the unpackaged CK group exhibiting the highest loss (*p* < 0.05). In contrast, OPEO/PVA films significantly mitigated weight loss, with the BPO-20 and BPO-15 groups recording the lowest rates (25.83% and 28.21%, respectively) by day 20. This performance is attributed to the high concentration of OPEO active compounds, which delay senescence, and the films’ ability to maintain a near-equilibrium humidity micro-atmosphere, effectively inhibiting transpiration [37].

Vitamin C (VC), a vital but oxidation-sensitive nutrient, serves as a hallmark of blueberry nutritional quality and shelf life [38]. As illustrated in Figure 4C, the VC content in all groups decreased progressively; however, the BPO-15 group exhibited the highest retention after 20 days, significantly outperforming the CK group (*p* < 0.05). This preservation is primarily due to the synergistic effect of the OPEO active components and the biomimetic structural barrier, which restricts gas exchange and minimizes the oxygen availability required for VC oxidation [39]. The results underscore that the integrated biomimetic architecture enhances the film’s antioxidant efficacy, thereby maximizing nutrient retention.

Blueberry decay is primarily driven by respiratory metabolism and microbial infection [40]. As shown in Figure 4D, decay rates in all groups rose over time, but the unpackaged CK group’s decay rate far exceeded that of the BPO-0 group by day 4 due to unsuppressed respiration and direct environmental exposure. In contrast, the biomimetic film groups maintained significantly lower decay rates by acting as a physical barrier. This protective efficacy is attributed to the cross-scale hierarchical gradient structure inspired by orange peels; its unique topography and high surface area inhibit microbial contact and infection, effectively extending the fruit’s shelf life [41].

Anthocyanin content, a key indicator of blueberry freshness and ripeness, reflects the intensity of fruit skin color [42]. As shown in Figure 4E, anthocyanin levels in all groups initially rose and then declined as storage progressed. During days 4–20, synthesis was insufficient to offset consumption, leading to an overall decrease. Notably, the BPO-15 group exhibited significantly superior anthocyanin retention (*p* < 0.05), reaching 0.73 mg/g at 20 days—a value significantly higher than other groups (*p* < 0.05). This indicates that the synergistic effect of the biomimetic structure and optimal orange peel essential oil dosage effectively preserves anthocyanins and maintains fruit freshness, thereby extending shelf life [43].

Soluble solids content (SSC), comprising sugars and organic acids, is a key freshness indicator [44]. During ripening, acids convert to sugars, increasing SSC; however, respiration-driven sugar consumption during senescence leads to an eventual decline [45]. As shown in Figure 4F, SSC in all groups initially rose—due to post-harvest ripening—and then decreased after 4 days of storage. All treatment groups effectively mitigated this decline compared to the CK group. By day 20, the BPO-20 (5.87%) and BPO-15 (6.2%) groups exhibited the best performance. These data suggest that the biomimetic films, through the synergistic action of the orange peel essential oil’s sustained release and the biomimetic surface structure, effectively inhibit respiration and metabolic rates, thereby preserving the SSC in blueberries [46].

Nevertheless, from the perspective of real-world food application, it must be noted that a comprehensive sensory analysis (encompassing taste, off-odor, and consumer acceptability) was not integrated at this stage, which represents a limitation. Traditional human sensory evaluations are frequently accompanied by inevitable subjective human variance and cognitive bias, which might compromise the strict reproducibility required for materials-oriented characterizations.

In our active packaging system, this potential aroma interference is assumed to be restricted. The moisture-induced sustained-release kinetics of the PVA hydrogel network successfully prevent an acute ‘flash burst’ of intensive essential oil vapors, and the natural citrus notes of D-limonene display excellent olfactory compatibility with the native volatile profile of ripening blueberries. To thoroughly eliminate subjective human errors and balance the paper’s focus, rigorous and objective instrumental flavor profiling via Electronic Nose (E-nose) coupled with gas chromatography will be prioritized in our forthcoming pilot-scaling phase to fully guarantee flavor integrity alongside extended shelf life.

## 3. Conclusions

The results of this study indicate that the OPEO/PVA bio-inspired film exhibits effective antioxidant activity and regulated antimicrobial properties. The PVA hydrogel network successfully encapsulates the active components of the orange peel essential oil while maintaining acceptable transparency and a favorable mechanical balance. The synergistic integration between the OPEO chemical barrier and the replicated surface topography contributes to stabilizing blueberry firmness, reducing postharvest decay, and mitigating nutrient loss. These findings demonstrate a promising conceptual strategy for developing active food packaging components utilizing natural biodegradable matrices. However, from the perspective of real-world implementation, multiple laboratory-scale constraints must be addressed prior to industrial commercialization; critical parameters such as mass-production scalability, precise volatile migration kinetics, long-term aging stability, and comprehensive sensory acceptance remain uninvestigated in this baseline stage. Consequently, future lines of work will be systematically directed toward evaluating these unexplored operational facets and establishing objective instrumental sensory matrices. This biomimetic structured material holds potential as a viable alternative for specialized fruit preservation scenarios, serving as a stepping stone toward advanced agricultural packaging configurations.

## 4. Materials and Methods

### 4.1. Materials and Instruments

Fresh oranges and fresh blueberries, purchased from Walmart in Changchun; orange peel essential oil, Jiangxi Jianmin Natural Fragrance Oil Factory (Ji’an, China); polyvinyl alcohol, Shaanxi Panlong Yihai Pharmaceutical Co., Ltd. (Xi’an, China); glycerol, Sinopharm Chemical Reagents Co., Ltd. (Shanghai, China); *Escherichia coli* and *Staphylococcus aureus*, Jilin Agricultural University Laboratory (Changchun, China); DPPH, ABTS, Sigma (St. Louis, MO, USA); anhydrous ethanol, Beijing Dingguo Changsheng Biotechnology Co., Ltd. (Beijing, China); silica gel, Beijing Dingguo Changsheng Biotechnology Co., Ltd. (Beijing, China); potassium bromide, Beijing Bailinwei Technology Co., Ltd. (Beijing, China); polydimethylsiloxane, Dow Corning 184, Dow Chemical Company (Midland, MI, USA); sodium hydroxide, Tianjin Zhiyuan Chemical Reagents Co., Ltd.; Phenolphthalein, Tianjin Zhiyuan Chemical Reagents Co., Ltd. (Tianjin, China); 2,6-dichloroisophenol, Tianjin Huasheng Chemical Reagents Co., Ltd. (Tianjin, China); sterile distilled water, Laboratory of Jilin Agricultural University (Changchun, China).

### 4.2. Experimental Methods

#### 4.2.1. Preparation of Biomimetic Films

Polydimethylsiloxane anti-structuring negative film:

After washing the freshly peeled orange peel with clean water and wiping it dry with anhydrous ethanol, the inner side of the dried orange peel was secured to a glass dish using double-sided tape. Polydimethylsiloxane (PDMS) and Dow Corning 184 curing agent were mixed in a 10:1 ratio and sonicated for 30 min to remove air bubbles; then, we pipetted 40 mL of PDMS and evenly coated the orange peel surface. We heated and dried it at 60 °C for 5 h. Once the solution on the orange peel surface had cured, we added distilled water; after 5 h, we removed and peeled off the peel to obtain a PDMS reverse-structure negative mold (SP-PDMS) of the biomimetic orange peel surface. Additionally, we applied PDMS to a glass slide surface as a blank control.

Bionic film:

We diluted orange peel essential oil 50-fold with 95% ethanol, homogenized it at 10,000 rad/s for 10 min, and let it stand for 30 min to obtain a uniform emulsion of orange peel essential oil. We prepared a sweet orange essential oil–polyvinyl alcohol (PVA) mixture solution in a specific ratio, poured it onto an SP-PDMS film, froze it at −20 °C for 10 h, and then thawed it at room temperature under 40% relative humidity for 4 h. After repeating this cycle five times, the film was peeled off to obtain a cationic film with a regular structure and an orange peel surface (OPEO/PVA biomimetic film). The peeled film was placed in an incubator at room temperature (22 ± 2 °C) and 40% relative humidity for 12 h. Preliminary experiments indicated that an excess of orange peel essential oil, due to its high lipophilicity, would disrupt the structural stability of the PVA gel network. This leads to precipitation after film formation and hinders the replication of the orange peel surface structure. Therefore, this study focused on biomimetic films containing 0%, 5%, 10%, 15%, and 20% orange peel essential oil, respectively, and named them BPO-0, BPO-5, BPO-10, BPO-15, and BPO-20.

#### 4.2.2. Microscopic Observation of the Surface Microstructure of a Natural Orange Peel and a Polydimethylsiloxane Anti-Structured Negative Film

We cut the orange peel and SP-PDMS films into 1 × 1 cm pieces, attached them to microscope slides using double-sided tape, and observed the microstructure of the sample surfaces under a stereomicroscope (SZX10, Olympus, Tokyo, Japan). The microscope features five standard objective magnifications: 4×, 10×, 16×, 25×, and 40×. The diopter adjustment range is ±5 diopters for both eyes, and the focus travel is 50 mm.

#### 4.2.3. Water Vapor Permeability

WVP was determined gravimetrically following the method of Mallick et al. [47]. Film samples were sealed over cups containing 30 g of silica gel and placed in a desiccator with saturated KNO_3_ solution (25 °C and 90% RH). The weight change (∆M) was monitored for 10 days until a steady state was reached. WVP (g m^−1^ s^−1^ Pa^−1^) was calculated using Equation (1):

(1) $$WVP=\frac{∆M\times d}{T\times A\times ∆P}$$

where d is bionic film thickness, A is effective area, T is time interval, and ∆P is the water vapor pressure differential.

#### 4.2.4. Swelling Degree and Solubility

SD and WS were measured according to Wu et al. [48] and Chen et al. [49]. Dried samples (M_1_ or M_0_) were immersed in 50 mL distilled water for 24 h at 25 °C. For SD, samples were weighed after removing surface water (M_2_). For WS, samples were re-dried to a constant weight (M_1_ after immersion). Calculations were performed as follows:

(2) $$SD\left(\%\right)=\frac{M_{2}-M_{1}}{M_{1}}\times 100\%$$

(3) $$WS\left(\%\right)=\frac{M_{1}-M_{0}}{M_{0}}\times 100\%$$

where SD (%) is the swelling degree; M_1_ and M_2_ are the masses (g) of the sample before and after soaking, respectively; WS (%) is the water solubility and M_0_ and M_1_ represent the dry masses (g) of the film before and after immersion, respectively.

#### 4.2.5. Microstructure and Chemical Composition

Surface Morphology: Film surfaces were gold-sputtered and observed using Scanning Electron Microscopy (SEM) at an accelerating voltage of 20 kV. Chemical Structure: Fourier Transform Infrared (FT-IR) spectra were recorded in the range of 4000–400 cm^−1^ at a resolution of 4 × 4 cm^−1^ using the KBr pellet method (64 scans). Crystalline Structure: X-ray diffraction (XRD) patterns were obtained (40 kV, 40 mA) from 2θ = 5° to 40° at a rate of 2°/min. The crystallinity index $X_{C}$ was calculated using Equation (4):

(4) $$X_{C}=\frac{A_{C}}{A_{C}+A_{a}}\times 100\%$$

where $A_{C}$ and $A_{a}$ represent the integral areas of crystalline peaks and amorphous halos, respectively.

#### 4.2.6. Optical Properties (Color and Opacity)

The surface color parameters (∆L, ∆a, ∆b) of the biomimetic films (8 × 8 cm) were measured using a colorimeter, with a standard white plate as a reference. The total color difference (∆E) was calculated according to Equation (5):

(5) $$∆E=\sqrt{{∆a}^{*2}+∆b^{*2}+∆L^{*2}}$$

In the formula: ∆a* = a* − a_0_;

∆b* = b* − b_0_;

∆L* = L* − L_0_.

The film opacity was determined by measuring the absorbance at 600 nm ($A_{600}$) using a UV–Vis spectrophotometer. Film samples (1 × 3 cm) were placed in the cuvette, and opacity (mm^−1^) was calculated using Equation (6):

(6) $$Opacity\left(mm^{-1}\right)=\frac{A_{600}}{d}$$

where $A_{600}$ is the absorbance at 600 nm and d is the film thickness (mm).

#### 4.2.7. Thickness and Moisture Content

Film thickness was determined using a digital micrometer (0.001 mm accuracy) at five random positions (center and four perimeter points), and the average value was recorded. The moisture content (MC) was determined gravimetrically according to the method of Liu et al. [50]. Film specimens (1 × 4 cm) were weighed (M_0_) and then dried in an electric forced-air oven at 105 °C for 12 h. The final weight (M_1_) was recorded, and MC was calculated as follows:

(7) $$MC\left(\%\right)=\frac{M_{0}-M_{1}}{M_{0}}\times 100\%$$

#### 4.2.8. Mechanical Properties

Tensile strength (TS) and elongation at break (EAB) were measured using an electronic universal testing machine following the method of Tan et al. [51]. Film specimens (5 × 1.5 cm) were mounted with an initial gauge length of 50 mm and stretched at a constant rate of 50 mm/min. TS (MPa) and EAB (%) were calculated according to Equations (8) and (9):

(8) $$TS\left(MPa\right)=\frac{F}{d\times W}$$

(9) $$EAB\left(\%\right)=\frac{L_{1}-L_{0}}{L_{0}}\times 100\%$$

where TS (MPa) and EAB (%) are tensile strength and elongation at break; F is the maximum force (N); d and W are the film thickness and width (mm); and L_0_ and L_1_ are the initial and fracture lengths (mm).

#### 4.2.9. Antioxidant Activity and Antibacterial Capacity

The antioxidant potential of the biomimetic films was evaluated via DPPH and ABTS radical scavenging assays according to Shekari et al. [52] and Roy et al. [53]. For the DPPH assay, film extracts (0.1 g in 10 mL 50% ethanol) were reacted with DPPH solution in the dark for 30 min, and absorbance was measured at 517 nm. For the ABTS assay, the film solution was mixed with a diluted ABTS cation solution and incubated for 30 min before measuring absorbance at 734 nm. The scavenging activities were calculated using Equations (10) and (11):

(10) $$DPPH=1-\frac{A_{1}-A_{2}}{A_{0}}\times 100$$

(11) $$ABTS\left(\%\right)=\frac{A_{control}-A_{sample}}{Acontrol}\times 100$$

where A_0_, A_1_, and A_2_ are the absorbance values of the blank control, the measured sample, and the sample control, respectively. A_control_ and A_sample_ are the absorbance values of the control and the film sample, respectively.

The antimicrobial efficacy against *Escherichia coli* and *Staphylococcus aureus* was assessed using the agar disk diffusion method and a bacterial adhesion assay [54]. In the disk diffusion test, the diameter of the inhibition zone was measured after 24 h of incubation at 37 °C. For the bacterial adhesion analysis, film samples were incubated with bacterial suspensions for 24 h, followed by serial dilution and colony counting on agar plates to evaluate the inhibitory effect of the films on bacterial attachment and growth.

#### 4.2.10. Blueberry Sample Processing

We selected fresh blueberries that were uniform in size, had consistent ripeness, and were free from pests, diseases, or external damage. After sterile washing and air-drying, we divided them into six groups of five berries each. Groups 1–5 were sequentially packaged using BPO-0, BPO-5, BPO-10, BPO-15, and BPO-20 films, respectively, and sealed with a plastic sealing machine. Group 6 (CK) served as the unpackaged control group. The packaged blueberries were stored at 4 °C for 20 days. Various indicators for each group were recorded and measured on the day of packaging, as well as at 4, 8, 12, 16, and 20 days.

#### 4.2.11. Blueberry Firmness and Weight-Loss Rate

The firmness of the blueberries was determined using a GY-3 fruit hardness tester (Zhejiang Topyunong Technology Co., Ltd., Hangzhou, China). The weight loss rate (WLR) was evaluated gravimetrically according to the method described by Ren et al. [55]. The mass of the blueberries was recorded every two days during the storage period, and the WLR (%) was calculated using Equation (12):

(12) $$WLR\left(\%\right)=\frac{M_{0}-M_{1}}{M_{0}}\times 100\%$$

where M_0_ is the initial mass of the blueberries before storage (g) and M_1_ is the mass measured at each sampling interval (g).

#### 4.2.12. Decay Rate

The decay rate (DR) of the blueberries was evaluated according to the method described by Ren et al. [55]. The fruits were classified into different decay grades (L) based on the severity of the rot. The DR (%) was calculated using Equation (13):

(13) $$DR\left(\%\right)=\sum \frac{L_{0}\times n}{L_{h}\times N}\times 100\%$$

where L is the decay grade, n is the number of blueberries in that grade, L_h_ is the highest decay grade, and N is the total number of blueberries in the treatment group.

#### 4.2.13. Vitamin C in Blueberries

Vitamin C content in blueberry fruits was determined using the 2,6-dichlorophenolindophenol titration method, with results expressed in mg/100 g [56].

#### 4.2.14. Blueberry Anthocyanins and Soluble Solids

Following the method established by Yang and colleagues [57] for measuring anthocyanins in blueberries, the pH differential method was employed to determine the anthocyanin content.

The soluble solids content (total soluble solids, TSS) in blueberries was determined using a refractometer (PAL-1, ATAGO, Tokyo, Japan) and expressed as a mass fraction (%).

#### 4.2.15. Statistical Analysis

Each experiment was conducted in triplicate. Data are expressed as “x ± s.” Graphs were generated using Origin 2021. Univariate analysis of variance (ANOVA) was performed using SPSS 26.0. Multiple comparisons were conducted to statistically analyze the significance of differences among means. Statistical significance was set at *p* < 0.05. All specimens for physical and mechanical characterizations were randomly sampled from three independent replication batches to verify the reproducibility of the fabrication process.

## Figures and Tables

**Table 1:** Color values of the bionic films.

Bionic Films	L*	a*	b*	ΔE*
BPO-0	90.55 ± 0.59 ^a^	−0.61± 0.02 ^a^	4.87 ± 0.06 ^d^	5.94 ± 0.10 ^e^
BPO-5	88.16 ± 0.43 ^b^	−1.03 ± 0.05 ^b^	5.12 ± 0.08 ^c^	7.34 ± 0.08 ^d^
BPO-10	85.95 ± 0.94 ^c^	−1.09 ± 0.05 ^bc^	5.20 ± 0.05 ^c^	8.20 ± 0.18 ^d^
BPO-15	85.03 ± 0.43 ^c^	−1.28 ± 0.04 ^d^	6.38 ± 0.11 ^a^	11.39 ± 0.84 ^b^
BPO-20	83.05 ± 0.25 ^d^	−1.17 ± 0.05 ^c^	6.19 ± 0.02 ^b^	10.19 ± 0.25 ^c^

Note: Data are expressed as mean ± standard deviation (*n* = 3). Different lowercase letters (a–e) within the same column indicate significant differences (*p* < 0.05) according to Duncan’s multiple range test.

**Table 2:** Crystalline and amorphous states and the crystallinity index of the bionic films.

Bionic Films	2θcr (deg)	2θam(deg)	FWHM (deg)	Xc (%)
BPO-0	19.80	21.50	0.82	44.26 ± 0.65
BPO-5	19.82	21.75	1.48	27.15 ± 0.48
BPO-10	19.85	22.10	2.65	13.42 ± 0.35
BPO-15	-	19.80	6.54	<3.0
BPO-20	-	19.80	7.15	<2.5

**Table 3:** Mechanical properties of biomimetic film.

Bionic Films	Tensile Strength (Mpa)	Elongation at Break (%)
BPO-0	5.34 ± 0.18 ^d^	152.93 ± 5.58 ^b^
BPO-5	6.69 ± 0.13 ^c^	174.32 ± 7.48 ^a^
BPO-10	7.21 ± 0.36 ^bc^	166.68 ± 2.82 ^a^
BPO-15	7.65 ± 0.22 ^b^	147.82 ± 3.25 ^b^
BPO-20	6.93 ± 0.19 ^c^	112.09 ± 7.77 ^c^

Note: Data are expressed as mean ± standard deviation (*n* = 3). Different lowercase letters (a–d) within the same column indicate significant differences (*p* < 0.05) among groups, determined by one-way ANOVA followed by Duncan’s multiple range test.

**Table 4:** Antioxidant activity and antibacterial capacity of different bionic films.

Bionic Film	Inhibition Zone Diameter (mm)		DPPH Radical Scavenging (%)	ABTS Radical Scavenging (%)
	*E. coli*	*S. aureus*		
OPEO	13.06 ± 0.14 ^a^	12.02 ± 0.27 ^a^	——	——
BPO-0	——	——	1.66 ± 0.48 ^e^	2.09 ± 0.60 ^e^
BPO-5	5.44 ± 0.20 ^e^	4.25 ± 0.21 ^e^	45.60 ± 1.20 ^d^	35.84 ± 0.81 ^d^
BPO-10	6.30 ± 0.25 ^d^	5.42 ± 0.06 ^d^	54.42 ± 1.33 ^c^	45.60 ± 0.79 ^c^
BPO-15	7.60 ± 0.19 ^c^	6.66 ± 0.23 ^c^	64.29 ± 1.12 ^b^	52.84 ± 0.70 ^b^
BPO-20	8.40 ± 0.39 ^b^	7.30 ± 0.17 ^b^	67.46 ± 0.56 ^a^	56.46 ± 0.67 ^a^

Note: Data are expressed as mean ± standard deviation (*n* = 3). Different lowercase letters (a–e) within the same column indicate significant differences (*p* < 0.05) among groups, as determined by one-way ANOVA followed by Duncan’s multiple-range test. “——” indicates not detected or not applicable.

## Data Availability

The original contributions presented in this study are included in the article. Further inquiries can be directed to the corresponding authors.
